# Utterance-Style-Dependent Speaker Verification Using Emotional Embedding with Pretrained Models

**DOI:** 10.3390/s25175284

**Published:** 2025-08-25

**Authors:** Long Pham Hoang, Hibiki Takayama, Masafumi Nishida, Satoru Tsuge, Shingo Kuroiwa

**Affiliations:** 1 Department of Computer Science, Faculty of Informatics, Shizuoka University, Hamamatsu Campus, 3-5-1 Johoku, Chuo-ku, Hamamatsu 432-8011, Japan; pham.hoang.long.21@shizuoka.ac.jp (L.P.H.); takayama.hibiki.19@shizuoka.ac.jp (H.T.); 2 Department of Information Systems, School of Informatics, Daido University, 10-3 Takiharucho, Minami-ku, Nagoya 457-0819, Japan; tsuge@daido-it.ac.jp; 3 Faculty of Informatics, Chiba University, Nishi-Chiba Campus, 1-33 Yayoicho, Inage-ku, Chiba 263-8522, Japan; kuroiwa@faculty.chiba-u.jp

**Keywords:** speaker verification, emotion-dependent authentication, score fusion, emotion embeddings, biometric authentication, user usability

## Abstract

**Highlights:**

**What are the main findings?**

- The score fusion-based emotion-dependent speaker verification system reduces the equal error rate to 1.13% with HuBERT, outperforming conventional methods.
- Compared to neutral speech, sadness is the optimal emotion for verification, offering low user burden, high score stability, and robust performance.

**What is the implication of the main finding?**

- Emotion-dependent verification enhances security over traditional methods, making it viable for applications requiring robust authentication.
- The effectiveness of grief suggests that emotional speech styles can be adapted to improve both the user experience and verification accuracy in biometric systems.

**Abstract:**

Biometric authentication using human physiological and behavioral characteristics has been widely adopted, with speaker verification attracting attention due to its convenience and noncontact nature. Conventional speaker verification systems remain vulnerable to spoofing attacks, however, often requiring integration with separate spoofed speech detection models. In this work, the authors propose an emotion-dependent speaker verification system that integrates speaker characteristics with emotional speech characteristics, enhancing robustness against spoofed speech without relying on additional classification models. By comparing acoustic characteristics of emotions between registered and verification speech using pretrained models, the proposed method reduces the equal error rate compared to conventional speaker verification systems, achieving an average equal error rate of 1.13% for speaker verification and 17.7% for the anti-spoofing task. Researchers additionally conducted a user evaluation experiment to assess the usability of emotion-dependent speaker verification. The results indicate that although emotion-dependent authentication was initially cognitively stressful, participants adapted over time, and the burden was significantly reduced after three sessions. Among the tested emotions (anger, joy, sadness, and neutral), sadness proved most effective, with stable scores, a low error rate, and minimal user strain. These findings suggest that neutral speech is not always the optimal choice for speaker verification and that well-designed emotion-dependent authentication can offer a practical and robust security solution.

## 1. Introduction

In recent years, biometric authentication has become increasingly familiar due to the development of technologies such as facial and fingerprint recognition and their practical applications. Among these, speaker verification, which uses voice as the authentication key, is attracting attention due to its cost-effectiveness (requiring only a single microphone) and low burden on the user due to its contactless approach. Speaker verification holds exciting potential for real-world applications, including user authentication for smartphones and smart speakers, as well as fraud detection in financial service call centers.

One downside of using speaker verification is the risk of different types of spoofing attacks. Among these, replay attacks, where a recorded voice is played back to deceive the system, are a major concern. This is because the spectrograms of playback audio can closely resemble those of genuine speech [1], increasing the false acceptance rate (FAR) in speaker verification systems, as demonstrated by Wu et al. [2].

Additionally, advancements in AI-driven speech synthesis, such as WaveNet-based neural vocoders [3], have enabled the creation of highly realistic synthetic voices [4], posing significant threats to system security. In one study, Yuan et al. [5] demonstrated an effective spoofing attack using a GAN-based text-to-speech model against i-vector-based speaker verification systems [6], which were highly potent at the time. These vulnerabilities highlight the urgent need for more robust speaker verification technologies [7].

To counter these vulnerabilities, research is currently being conducted on spoofed speech detection to strengthen the robustness of speaker verification systems. By incorporating a model that distinguishes natural human speech from synthesized or duplicated speech in the first stage of a speaker verification system, the FAR has been significantly reduced [8,9]. The ASVspoof 2021 competition [9] revealed its limitations, however, with equal error rates (EERs) of 24.25% for the physical access task (replay attacks) and 20.63% for the deepfake task (spoofed speech spoofing). These results indicate that current spoofed speech detection models are insufficient to fully secure speaker verification systems.

An alternative approach to improving robustness is the speaker verification method using singing voices, as proposed by Moriyama et al. [10]. Their study used singing voices for authentication, achieving a notable FAR reduction by leveraging pitch and speaker identity as dual authentication keys, provided the correct pitch remained undisclosed to impostors. These findings suggest that introducing novel authentication methods and speech types, especially those difficult for machines or speech synthesis models to replicate, could significantly enhance the robustness of speaker verification systems without requiring integration with spoofed speech detection models. Research exploring such innovative verification methods and speech styles remains limited, however.

To address this gap, researchers propose an emotion-dependent speaker verification framework that aims to develop a standalone system inherently resilient to spoofed speech, eliminating the need for separate spoof detection models. While conventional speaker verification systems often overlook the role of emotions in voice, with some studies even employing techniques to mask and eliminate emotional information, normalizing speech to a neutral state to focus solely on speaker identity [11]. This approach may inadvertently reduce robustness against spoofing attacks, as it ignores the unique emotional nuances in the speech. In contrast, our research leverages emotional speech characteristics as a primary authentication key, enhancing system performance while making it harder for spoofed audio to succeed due to the challenge of accurately reproducing emotional variations. In this framework, users preregister specific emotional characteristics of their speech during enrollment and must reproduce similar characteristics during verification. By requiring speakers to demonstrate both their voice characteristics and the registered emotional speech characteristic for verification, which is significantly more challenging for impostors than replicating voice alone, this framework is expected to enhance security compared to conventional approaches that use only voice information.

Additionally, unlike traditional emotion verification systems that classify specific emotions, the proposed framework compares the acoustic characteristics of emotions expressed in speech during registration and verification to determine their similarity. This comparison-based method does not require users to precisely replicate genuine, natural emotions but instead relies on speakers’ voluntarily induced emotional characteristics elicited through simple prompts. This approach not only makes it easier for users to produce speech with specific emotional qualities but also ensures reproducibility without complex stimulation protocols, enhancing both usability and robustness.

For implementing this emotion-dependent speaker verification framework, researchers explored two approaches: (1) a two-stage verification process, comprising emotion verification followed by speaker verification, and (2) a score-integration method that combines speaker and emotion embeddings. Researchers then compared these approaches to identify the optimal model for speaker verification performance, evaluated through metrics such as EER. Subsequently, the best-performing model was evaluated for robustness against a dataset of spoofed speech, including spoofed speech generated by a voice conversion model, to validate its effectiveness in countering spoofing attacks.

Finally, because emotion-dependent speaker verification requires users to perform emotional speech for authentication, it may impose a greater physical burden on users compared to traditional speaker verification methods. The extent of this additional burden remains unclear. To address this limitation, researchers conducted user evaluation experiments to explore the perceived effort of producing emotional utterances. A small-scale experiment was then conducted based on data collected from participants to investigate whether the proposed method could effectively compare the acoustic characteristics of voluntarily expressed emotions under normal conditions, further validating its robustness and applicability in real-world scenarios.

## 2. Materials and Methods

This study proposes an emotion-dependent speaker verification system that enhances robustness against spoofing attacks by comparing acoustic characteristics of emotions expressed in the speech between registered and verified speakers. The system employs two approaches: a two-step verification method and a score fusion-based method, both utilizing x-vectors extracted via the ECAPA-TDNN model. Below, researchers describe the system workflow, feature extraction, and evaluation process, focusing on the rationale for key methodological choices.

The emotion-dependent speaker verification system operates in two configurations. In the two-step approach, the system first verifies whether the input speech matches the user’s preregistered emotional characteristics using x-vectors. If accepted, it then confirms the speaker’s verification using the same x-vector framework. In the score fusion approach, speaker and emotion embeddings are extracted from input speech, and their cosine similarity scores are combined into a single integrated score using a weighted sum. This integrated score is compared against a threshold to authenticate the user, offering a streamlined alternative to the two-step method.

### 2.1. DataSet

For model training and evaluation purposes, researchers employed the Japanese Twitter-based Emotional Speech (JTES) corpus [12], which comprises 20,000 Japanese utterances collected from 100 native Japanese speakers evenly distributed across gender (50 male, 50 female). The corpus includes four distinct emotions: anger, joy, sadness, and neutral, with each speaker contributing 50 sentences per category. All recordings are approximately five seconds in duration and are recorded at a 48 kHz sampling rate and 16-bit quantization. A key strength of the JTES corpus lies in its text-independent nature, as the utterances feature diverse linguistic content sourced from Twitter posts. This diversity is meant to enable robust emotion recognition and speaker authentication functions without relying on specific language patterns or predefined textual structures.

Table 1 shows examples of the speech content of each emotion as translated into English.

**Figure 1:**
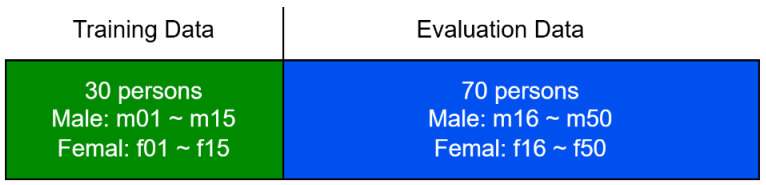
Experimental data segmentation.

Finally, all speech was downsampled to 16 kHz, and the data set was divided into 30 speakers for training and 70 speakers for evaluation; the gender balance is shown in Figure 1 below.

### 2.2. Two-Step Speaker Verification Approach

**Figure 2:**
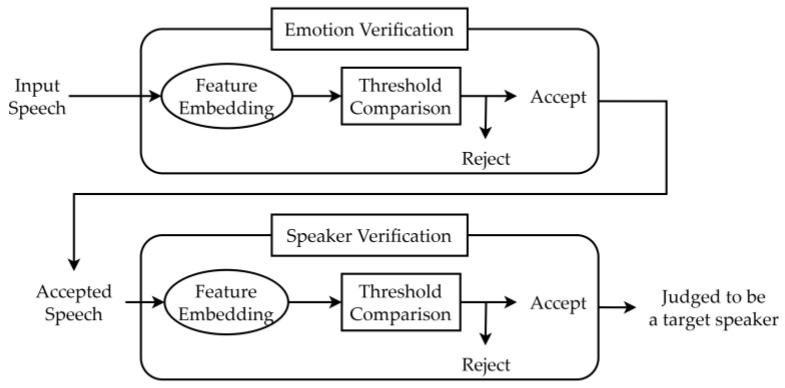
Emotion-dependent speaker verification system using a two-step approach.

Figure 2 shows a schematic diagram of the two-step speaker verification method. As explained above, the two-step approach prioritizes rejecting impostor speech by validating both emotional characteristics and speaker identity. For emotion verification, the authors extract 192-dimensional x-vectors using the pretrained ECAPA-TDNN model (Spkrec ECAPA VoxCeleb, SpeechBrain [13,14]), chosen for its ability to capture both speaker-specific and emotional speech characteristics, as supported by Pappagari et al. [15]. Unlike typical emotion recognition, which accepts a target emotion across speakers, our system accepts only the target speaker’s registered emotion (e.g., anger) while rejecting all impostor speech, regardless of emotion. Researchers acknowledge that x-vectors, while optimized for speaker verification, may have limitations in representing emotional information. To address this, two complementary methods are explored to evaluate their effectiveness in emotion verification.

- Threshold-based comparison: Cosine similarity between x-vectors of input and registered speech is computed and compared against a global threshold per emotion. Global thresholds were chosen to simplify system design and ensure consistency across users, balancing ease of implementation with performance. Speaker-specific thresholds, while potentially more accurate, require complex calibration, which researchers deferred for future work.
- Support vector machine (SVM)-based classification: A support vector machine (SVM) with a radial basis function kernel is trained on x-vectors to distinguish the registered emotion from others. This method was selected to leverage SVM’s strong discriminative power for binary classification, addressing the limitations of x-vectors in emotion representation noted by Pappagari et al. [15], and complementing the simplicity of the threshold-based approach.

For both methods, researchers utilized 100 speakers from the JTES corpus, selecting one speaker at a time as the target speaker, with the remaining 99 speakers serving as impostor speakers. This process was repeated 100 times, with each speaker acting as the target speaker once to compute average performance metrics.

For threshold-based verification, a registered x-vector was computed by averaging x-vectors from 10 utterances of the target emotion. Researchers then compared test utterance x-vectors to the registered x-vector using cosine similarity for emotion classification.

A single threshold was established for each emotion, shared across all registered speakers, resulting in four thresholds. To set each threshold, researchers used cosine similarity scores computed from a dataset of 4000 utterances of the registered emotion (100 speakers × 40 utterances) and 12,000 utterances of unregistered emotions (100 speakers × 3 emotions × 40 utterances). These thresholds were then optimized to achieve the EER, where the false rejection rate of the registered emotion (FRR-TE) equals the false acceptance rate of unregistered emotions (FAR-FE), using a validation subset. Finally, researchers applied the thresholds to evaluate FRR-TE and FAR-FE for impostor speakers on a separate test set comprising 40 utterances per emotion.

In the SVM-based emotion verification, researchers trained the SVM with a radial basis function kernel for each target speaker using 40 utterances: 10 of the registered emotion and 30 of the nonregistered emotions (10 per emotion). Hyperparameters (C and gamma) were tuned via fivefold cross-validation to maximize classification accuracy. The trained SVM was used to classify test utterances from both the target speaker and the 99 impostor speakers. Researchers averaged FRR-TE and FAR-FE across the 100 target speakers for target results and across 9900 impostor evaluations (99 impostors × 100 targets) for impostor results.

For speaker verification, the same ECAPA-TDNN model was used to extract x-vectors, with 10 utterances per emotion averaged to generate a registered x-vector. Cosine similarity scores were then compared against a threshold optimized for Equal Error Rate (EER), balancing the false rejection rate of the target speaker’s registered emotion and the false acceptance rate of impostor utterances. This evaluation was conducted using 70 speakers (2800 genuine utterances and 966,000 impostor utterances) in a leave-one-out strategy.

### 2.3. Score Fusion-Based Approach

The score fusion-based approach represents a novel contribution by integrating speaker and emotion verification into a single, streamlined decision process. This addresses the limitations of the traditional two-step method, in which the failure of either stage can compromise the overall performance. While the two-step approach may enhance speaker verification accuracy by incorporating emotion verification, its sequential nature can reduce both efficiency and robustness. To overcome these challenges, a score fusion strategy was employed to combine speaker and emotion information into a unified similarity score, enabling more reliable and efficient verification performance.

**Figure 3:**
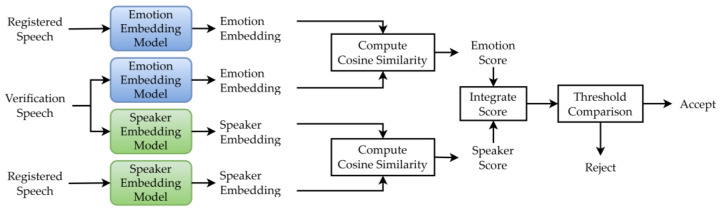
Emotion-dependent speaker verification system using score integration.

As shown in Figure 3, the proposed system extracted speaker and emotion embeddings from input speech and computed cosine similarity scores between these embeddings and those of the registered speech, yielding speaker and emotion verification scores. For each registered speaker, a registered speaker and emotion embedding were computed as the average of x-vectors from 10 utterances of the specified emotional style. These scores were combined into an integrated score using a weighted sum, as expressed below:

(1) $$integrated\;score=\alpha sim\left(s_{t},s_{r}\right)+\left(1-\alpha \right)sim\left(e_{t},e_{r}\right)$$

where α is a weighting factor, $s_{t}$ and $e_{t}$ represent the speaker and emotion embedding vectors for the test utterance, respectively, and $s_{r}$ and $e_{r}$ denote those for the registered utterance. The speaker verification score is then computed as $sim\left(s_{t},s_{r}\right)$, where $sim\left(e_{t},e_{r}\right)\;$ denotes the emotion score, with  sim() is the cosine similarity function. Finally, if the integrated score exceeded a predefined threshold, the user was authenticated; otherwise, they were rejected.

For speaker embeddings, researchers used the ECAPA-TDNN model [13,14]. For emotion embeddings, HuBERT [16] and wav2vec 2.0 [17] were evaluated in addition to the ECAPA-TDNN model. In the two-stage verification method (Section 3.1), only ECAPA-TDNN was used for emotion verification due to its established effectiveness with x-vectors for speaker verification. To explore more advanced emotion embeddings in the score fusion-based method, researchers evaluated ECAPA-TDNN alongside HuBERT and wav2vec 2.0, as these models have shown promise in emotion recognition with some fine-tuning [15,18]. The HuBERT model used was the Japanese-HuBERT-base from Rinna, wav2vec 2.0 was the Japanese-wav2vec2-base from Rinna, and ECAPA-TDNN was the Spkrec Ecapa Voxceleb model from SpeechBrain [13,14].

**Figure 4:**
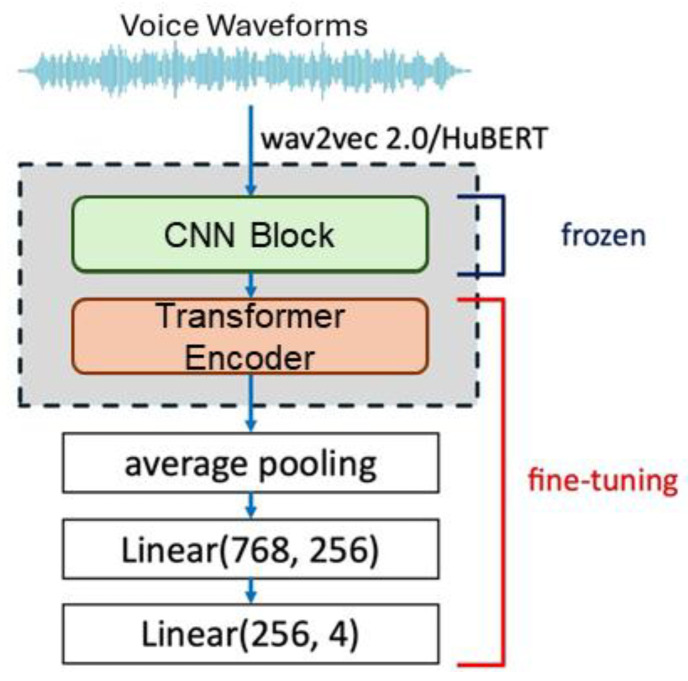
wav2vec 2.0/HuBERT fine-tuning.

For training, researchers fine-tuned three emotion embedding models using 3600 utterances from 30 speakers across four emotions (anger, joy, sadness, and neutral) from the JTES corpus, with 1200 utterances reserved for validation. For wav2vec 2.0 and HuBERT analysis, the 768-dimensional transformer output was averaged across frames, passed through a 256-dimensional linear layer, and fed into a four-class emotion classification layer, with the transformer and linear layers fine-tuned (Figure 4). For ECAPA-TDNN analysis, the output layer was modified for four-class emotion classification, and all model parameters were fine-tuned. Hyperparameters included cross-entropy loss, the Adam optimizer, a learning rate of 0.0001 for ECAPA-TDNN, and 0.000001 for wav2vec 2.0/HuBERT, batch sizes of four and 16, respectively, and 10 epochs.

For evaluation, utterances from 70 speakers in the JTES corpus were employed. Following a leave-one-speaker-out strategy, 70 evaluation rounds were conducted, ensuring that every speaker was tested as the target once. The final evaluation dataset comprised 2800 genuine utterances (70 speakers × 40 utterances of the registered emotion) and 966,000 impostor utterances (69 impostors × 4 emotions × 50 utterances × 70 target speakers). Finally, the threshold was optimized using the same cosine similarity-based approach as in two-stage speaker verification.

### 2.4. Anti-Spoofing Experiment

Following the performance comparison between the two-stage verification and score-fusion approaches, an anti-spoofing experiment was conducted to assess the robustness of the proposed emotion-dependent model against spoofing attacks.

In this experiment, spoofed speech samples were generated using seed-VC [19], a zero-shot voice conversion model, to imitate the voices of target speakers. The evaluation dataset consisted of 2800 genuine utterances (70 speakers × 40 utterances each) and 14,000 spoofed utterances (70 speakers × 4 emotions × 50 utterances per emotion). Spoofed utterances were created by converting speech from randomly selected non-registered speakers within the JTES corpus to mimic the voices of the target speakers.

The baseline system, which used only speaker embeddings extracted by ECAPA-TDNN, was compared to the proposed score-fusion model incorporating HuBERT emotion embeddings, which demonstrated better performance than the two-step verification approach (detailed results are provided in Section 3). Thresholds for each emotion were determined based on cosine similarity scores, optimized to minimize the equal error rate.

### 2.5. User Evaluation Experiment

As explained above, the emotion-dependent system requires users to perform emotional acting during authentication, which potentially increases the cognitive burden compared to conventional speaker verification. To assess this potential burden and evaluate the system’s usability, researchers conducted a user evaluation experiment focused on the relative effort involved in producing emotional utterances across four emotions (anger, joy, sadness, and neutral).

Eight male volunteers participated in the experiment, performing verification tests twice weekly over three weeks, with each test involving five utterances per emotion. Importantly, all participants were university students with no prior training or experience in acting or in consciously expressing emotions through speech. This ensured that the emotional expressions captured in the dataset were spontaneous and unrefined, reflecting how typical users might behave in real-world applications.

Tests were conducted in a quiet room with a single participant, using a SONY ECM-673/9X microphone, the STEINBERG UR22mkII audio interface, and a MacBook Air (M1, 2020). Audio was recorded at 16 kHz using Python (version 3.10.1) with the sounddevice (version 0.4.7) and wave libraries. The registered utterances were then recorded before the first verification session and included 10 utterances per emotion, with participants selecting either the provided sentences or self-selected sentences that promoted the four emotional expressions. The utterance content of each emotion is listed in Table 2 below.

Additionally, to evaluate whether the proposed system could accurately compare emotional speech characteristics between the registered and test utterances produced by non-professional speakers, an emotion verification experiment was conducted. Building on the experimental results provided in Section 3, researchers used HuBERT to extract emotion embeddings from the test utterances and compared them with the registered embeddings from the same participant using cosine similarity. An average similarity score ranging from −1 to 1 was computed shared across all 8 participants for each emotion, where a score closer to 1 indicated high emotional consistency between the registered and test utterances, while a score closer to −1 indicated strong emotional dissimilarity.

Finally, post-test questionnaires collected data on the perceived burden, with six responses per participant. The questionnaire items were these:

1. Relative burden of each emotion compared to neutral (scale: −3 to +3, mandatory).
2. Reasons for perceived burden (optional).
3. Willingness to use a conventional verification system (speaker verification only) daily (5-point scale, mandatory).
4. Willingness to use the emotion-dependent system daily, noting its enhanced security (5-point scale, mandatory).

## 3. Experimental Results

### 3.1. Two-Step Speaker Verification Method

The results of this approach are presented in three parts: emotion verification only, speaker verification only, and the integrated proposed method.

For emotion verification, Table 3 presents the experimental results of threshold-based emotion verification, and Table 4 shows those using SVM. In both Table 3 and Table 4, FRR-TE refers to the false rejection rate of the target emotion, and FAR-FE (false emotion) denotes the false acceptance rate of the nontarget emotion.

According to Table 3 and Table 4, the FRR-TE and FAR-FE values for impostors’ utterances indicate that the emotion verification process dismissed most utterances, whether expressing the target emotion or a nontarget emotion. In the threshold-based approach, impostor utterances exhibited a 99.00% FRR-TE for the registered emotion and a 0.00% FAR-FE for unregistered emotions, while in the SVM-based approach, the rates are 87.31% and 7.60%, respectively. These results underscore the high robustness of both methods in rejecting imposters.

Conversely, the FRR-TE for the target speaker’s utterances reveals a high FRR-TE for registered emotion and a notable FAR-FE for nonregistered emotions. Specifically, in the threshold-based approach (Table 3), the average FRR-TE for target speakers reached 32.29%, and FAR-FE was 32.33%, highlighting challenges in accurately verifying the target speaker’s emotions compared to the robust rejection of impostor speakers’ utterances. This high false rejection rate, particularly for genuine users, stems from x-vectors being optimized for speaker recognition rather than emotion detection.

In addition to comparing the results in Table 3 and Table 4, the threshold-based method outperforms the SVM-based method with lower FRR-TE for genuine speakers and FAR-FE for imposters. In the results of the integration of emotion verification and speaker verification described below, researchers therefore employed the method using threshold comparison for emotion verification due to its superior performance.

**Figure 5:**
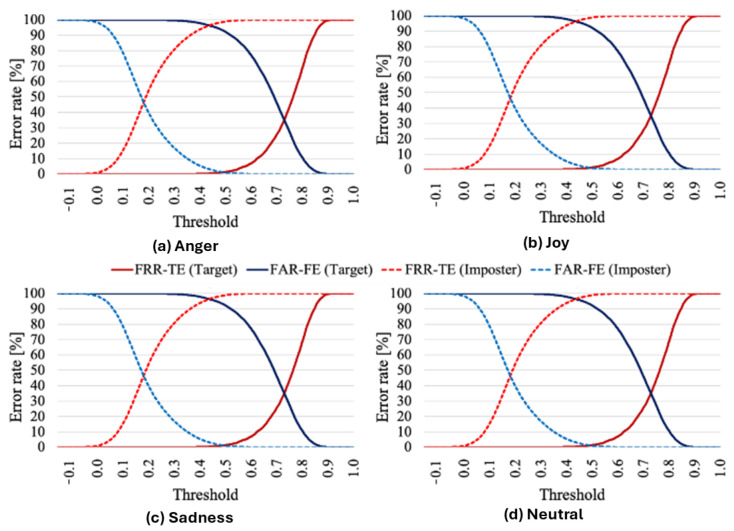
Error rates of emotion verification by threshold comparison.

Next, Figure 5 illustrates the error rates at various thresholds for emotion verification using the threshold-based approach. In Figure 5, FRR-TE (target) represents the false rejection rate of the target speaker’s registered emotion, FAR-FE (target) denotes the false acceptance rate of the target speaker’s nonregistered emotions, FRR-TE (impostor) indicates the false rejection rate of the impostor’s registered emotion, and FAR-FE (impostor) reflects the false acceptance rate of the impostor’s nonregistered emotions. The optimized threshold, used to compute the EER, is shown at the intersection of the blue and red lines in the figure.

Analysis of Figure 5 reveals that impostor speakers’ utterances were consistently rejected more frequently than those of target speakers across all thresholds and suggests that x-vectors capture more speaker-specific information than emotional information, facilitating effective differentiation between target and impostor speakers.

Next, Table 5 presents the results of speaker verification only. The data indicate that speaker verification using x-vectors achieves high accuracy across all emotions, with anger and sadness outperforming neutral emotions, suggesting that neutral speech is not necessarily optimal for speaker verification. Moreover, the EERs are consistently low, confirming the effectiveness of x-vectors for speaker identity verification.

**Figure 6:**
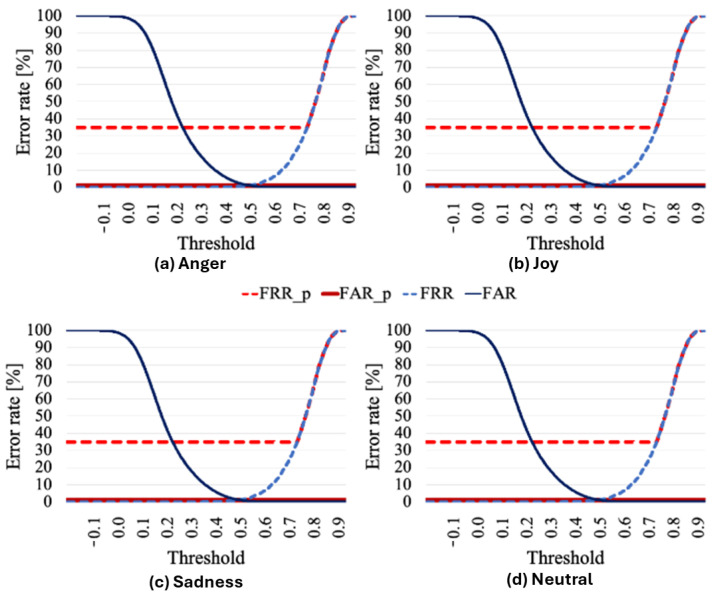
Results of proposed method and conventional method without emotion verification.

Finally, Figure 6 illustrates the error rates at various thresholds for conventional speaker verification without emotion verification and for the proposed method in which the first stage is emotion verification. In Figure 6, FRR_p and FAR_p represent the false rejection rate and false acceptance rate of the proposed method, respectively, while FRR and FAR denote those of the conventional method. As shown in Figure 6, the integrated system significantly reduces the FAR rate compared to conventional speaker verification, achieving near-zero FAR across all thresholds due to the emotion verification stage rejecting most impostor utterances.

The FRR increases due to the low accuracy of emotion verification, however, leading to more frequent rejections of genuine users and consequently elevating the EER.

Building on this, to address this limitation and further evaluate the potential of emotion verification, researchers adopted an integrated score-based method combining emotion and speaker verification, as described in the previous section.

### 3.2. Score Fusion-Based Method

Table 6 presents the EERs for the score fusion-based approach, which integrates emotion and speaker verification scores, compared to the conventional method relying solely on speaker verification scores. The proposed score fusion method consistently achieved lower EERs across all emotions compared to the conventional method, with HuBERT yielding the best performance, achieving an average EER of 1.13% at α = 0.9. Additionally, Table 7 below displays the EER for various values of weight α in the proposed method, using HuBERT for emotion verification. Varying α in Table 7 identified an optimal value of 0.9, with lower values increasing the EER. This optimal α = 0.9 reflects the greater variability in speaker identity (70 classes in the JTES corpus) compared to emotional categories (4 classes). Speaker features, derived from diverse vocal characteristics (e.g., pitch, timbre), exhibit higher natural variation across individuals, providing a stronger basis for discrimination. In contrast, emotional categories, while distinct, have less inter-class variability due to shared acoustic patterns within each emotion. The choice of optimal α may also be influenced by the diversity within the dataset, however. Specifically, the JTES corpus features emotional speech performed by experienced acting professionals, which may exaggerate emotional distinctions and influence the weighting toward α = 0.9. Therefore, this value may vary when applied to different datasets or real-world scenarios, where natural, untrained emotional expressions can introduce greater variability. Further research is needed to validate and adjust the α parameter across diverse datasets to ensure adaptability in practical applications.

Further analysis of Table 6 reveals distinct performance variations across emotions, with sadness (EER 0.82%) and anger (0.88%) outperforming neutral (1.05%) and joy (1.75%) in the score fusion-based method using HuBERT. This suggests that certain emotional speech characteristics may enhance the discriminability of speaker verification. The superior performance of sadness and anger may be attributed to their pronounced acoustic features, such as increased pitch variability and intensity in anger or slower tempo with lower pitch in sadness, providing better cues for distinguishing individual speakers compared to the more uniform characteristics of neutral speech. This finding highlights the benefits of leveraging emotional diversity, rather than suppressing it, offering improved robustness for speaker verification systems.

Collectively, these results demonstrate that the proposed score fusion-based method effectively mitigates the inaccuracy of emotion verification, thereby reducing the FRR. These findings highlight the potential of integrating emotion and speaker verification to achieve authentication systems that are both robust and accurate.

**Figure 7:**
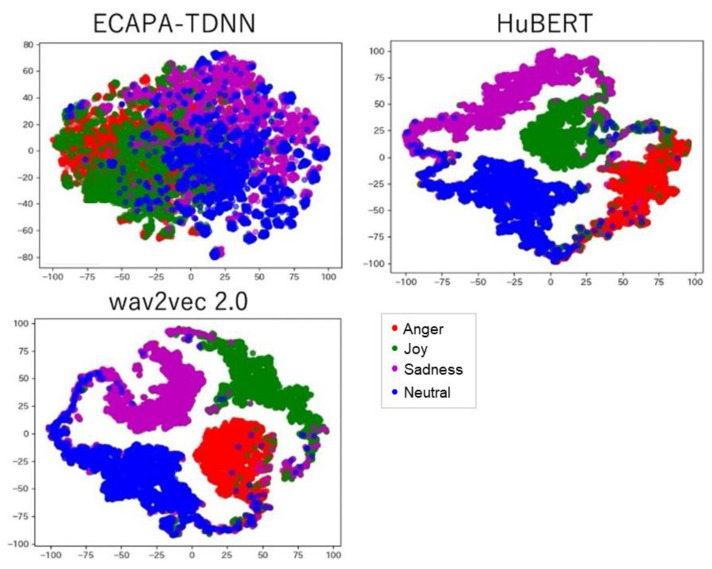
Distribution of each emotion embedding by dimensionality reduction using t-SNE.

In addition, to compare the effectiveness of three emotion verification models, researchers employed t-SNE for visualization after reducing the dimensionality of each extracted emotion embedding. The t-SNE visualization of emotion embeddings (Figure 7) revealed that HuBERT forms the most distinct emotion clusters, which contributes to its superior performance in the score fusion-based method by enhancing emotion separability in the integrated score.

### 3.3. Anti-Spoofing Experimental Results

In this experiment, a test was conducted to evaluate the robustness of the emotion-dependent speaker verification system against spoofed speech. Based on previous results showing that the score-fusion approach achieved an average EER of 1.13%, outperforming the two-step verification method, researchers selected it as the primary model for further comparison. Its robustness was assessed against a conventional speaker verification system that relied solely on speaker embeddings (ECAPA-TDNN).

Table 8 presents the EERs for both the score-fusion-based system and the conventional system using only speaker embeddings, while Table 9 shows the false acceptance rates for false emotion (FAR-FE) of these models. As shown in the tables, the conventional system yielded an EER of 25.1% and a false acceptance rate (FAR) of 17.6% when evaluated with spoofed utterances generated by voice conversion. In contrast, the proposed emotion-dependent system significantly reduced the EER to 17.7% and the FAR to 1.5% at α = 0.8.

Notably, sadness exhibits the lowest EER at 14.2%, reinforcing its robustness against spoofed audio, likely due to the distinct acoustic features such as slower tempo and lower pitch, which are challenging for synthesis models to replicate accurately. In contrast, anger shows a higher EER of 18.7%, an increase from its performance in non-spoofed scenarios (0.88% with α = 0.9), suggesting that the heightened pitch variability and intensity associated with anger may be more susceptible to imitation by advanced spoofing techniques. The conventional method, with EERs ranging from 19.1% (sadness) to 28.5% (joy), highlights its vulnerability, particularly with neutral speech (25.1%), which lacks the emotional nuances that enhance discrimination. These results affirm that the score fusion-based approach, by integrating emotional characteristics, significantly enhances resilience against spoofed attacks, with sadness emerging as the most effective emotional state, despite the unexpected rise in anger’s EER.

These results demonstrate the enhanced robustness of the emotion-dependent system in rejecting spoofed speech, particularly when the spoofing involves emotions not encountered during registration. Among all tested emotions, sadness proves to be the most effective in detecting spoofing speech. This improvement can be attributed to the additional requirement of verification of not only speaker identity but also the emotional characteristics of the registered utterance, which spoofed voice synthesis systems still struggle to replicate due to their limited capacity to model speaker-specific emotional expression.

### 3.4. User Evaluation Experimental Results

**Figure 8:**
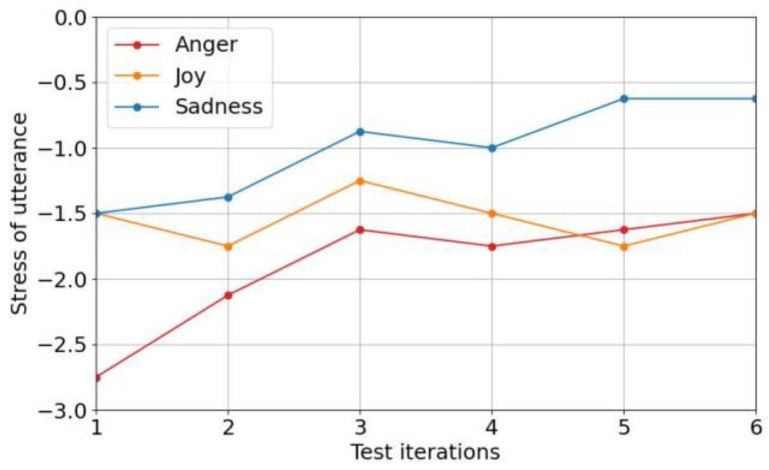
The burden of each emotional utterance.

Following the evaluation of emotion verification models, Figure 8 illustrates the perceived effort of emotional utterances across multiple tests, based on responses to Question 1 of the post-survey, with the effort level of neutral utterances set to zero. This figure shows that the perceived effort of emotional utterances decreased over time, with significant reductions by the third test as participants adapted to expressing emotions vocally. Sadness required the least effort, often comparable to neutral due to its tonal similarity to neutral speech. In contrast, anger and joy required greater vocal energy, resulting in higher perceived effort, particularly for anger, which some participants rarely expressed vocally.

Building on the effort analysis in Figure 8, researchers examined participant responses to understand the perceived effort of emotional speech. Participants noted that expressing emotions via their voices was somewhat difficult at the beginning due to unfamiliarity, particularly with emotions that demanded significant physical energy, such as anger and joy. Researchers did observe positive adaptations over time, however. Many participants reported that they became more comfortable with the task as well as finding it easier to express emotions such as joy and sadness. For instance, participants noted that joy was easier to express when raising their tone and that sadness was less burdensome due to its similarity to neutral speech. This adaptation suggests that the emotion-dependent system is most effective with continuous use; occasional gaps in practice led some participants to forget their expression techniques.

**Figure 9:**
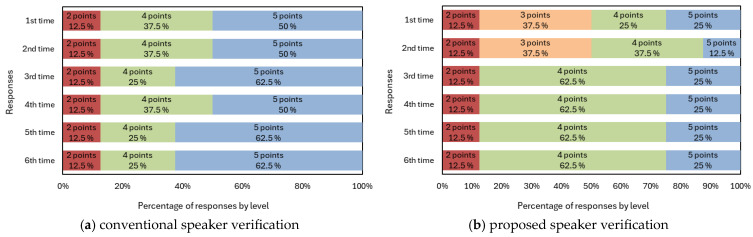
Subjective evaluation of the use of conventional and proposed speaker verification.

To further explore users’ experiences, Figure 9 presents results from a five-point rating scale (1 = very uncomfortable, 5 = very comfortable) assessing participants’ comfort with two systems: (a) a neutral-based speaker verification system (conventional speaker verification system) using neutral speech and (b) an emotion-dependent speaker verification system. Most participants expressed favorable views toward the neutral-based system, and approval ratings increased over time. In contrast, the emotion-dependent system initially received mixed feedback, with positive and negative opinions evenly divided in the first and second tests. Despite participants’ initial impressions, positive feedback increased significantly from the third test onward, reflecting adaptation to expressing emotions vocally.

**Figure 10:**
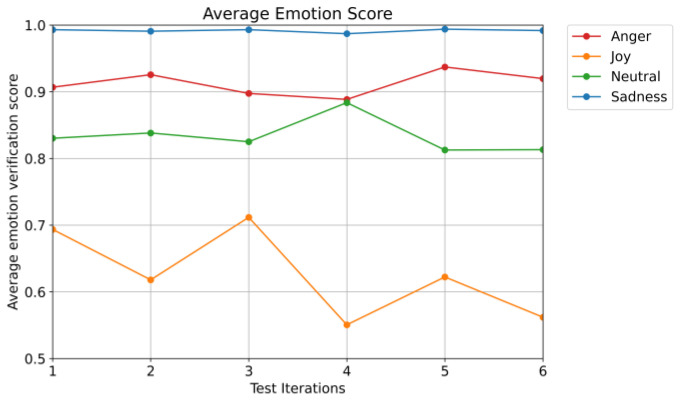
Average emotion verification scores across six test iterations for each emotion.

Finally, researchers evaluated the effectiveness of the emotion-dependent verification system in identifying emotional consistency between registered and test utterances collected from participants. Figure 10 presents the average emotion verification scores across six test iterations for four emotions (anger, joy, neutral, and sadness), based on cosine similarity.

As illustrated in the figure, sadness consistently achieved the highest average verification scores, remaining close to 1.0 throughout all tests. This suggests strong emotional consistency between registered and test utterances. Anger also maintained relatively high scores, though with slight fluctuations between tests, reflecting some difficulty in consistently reproducing intense emotions. Neutral speech showed stable performance with scores above 0.8, reflecting moderate consistency. In contrast, joy yielded the lowest and most variable scores, with a noticeable decline in later test iterations.

Despite having the lowest scores, joy’s average remained above zero (>0.5), indicating that all emotions, including the more challenging ones, were correctly recognized by the system. This result demonstrates that even spontaneous or self-directed emotional expressions can still contribute meaningfully to overall speaker verification accuracy.

These findings align with participant feedback, which noted that joy and anger were more difficult to express due to their energetic vocal demands. However, a slight improvement in verification scores, especially for anger, by the fifth iteration indicates a learning effect, with participants becoming more comfortable and consistent in expressing emotions vocally over time.

Taken together with the perceived effort and comfort analysis, these results show that while the emotion-dependent verification system introduces a higher cognitive and vocal burden initially, it can support effective and robust authentication as users adapt to emotional expression. Importantly, the experiment highlights that spontaneously expressed, non-acted emotions, rather than exaggerated or professionally acted ones, are sufficient for accurate verification, thereby reinforcing the system’s practicality for real-world deployment without requiring professional-level training in emotional speech.

## 4. Discussion

The results of this experiment provide important insights into the performance and usability of the proposed emotion-dependent speaker verification system, highlighting both its strengths and areas for improvement.

The two-step verification method, which integrates emotion verification with speaker verification, demonstrates robust performance in rejecting impostor utterances, as evidenced by the high FRR-TE (99.99% for threshold-based, 87.31% for SVM-based) and low FAR-FE (0.00% for threshold-based, 7.60% for SVM-based) for impostors’ utterances (Table 3 and Table 4). This indicates that both threshold-based and SVM-based emotion verification effectively distinguish impostor speakers, leveraging x-vectors’ strength in capturing speaker-specific information. The high FRR-TE (32.29% for threshold-based, 38.43% for SVM-based) for target speakers’ utterances reveals challenges in accurately verifying genuine speakers’ emotions, however. This discrepancy likely stems from x-vectors being primarily designed for speaker verification rather than emotion verification. In comparison, the threshold-based approach outperformed the SVM-based method, achieving lower FRR-TE and FAR-FE. Figure 5 further supports this, showing that impostor utterances are consistently rejected across thresholds, while target speakers face higher error rates due to the limited emotional information captured by x-vectors.

Additionally, the results from Table 5 indicate that speaker verification alone achieves high accuracy (average EER of 1.60%), with emotions such as anger and sadness outperforming neutral speech. This suggests that emotional speech may carry richer speaker-specific information than neutral speech, challenging the assumption that neutral speech is optimal for speaker verification.

The integration of emotion verification in the two-step method increases the FRR for genuine users due to the lower accuracy of emotion verification, however, elevating the overall EER (Figure 6). This trade-off highlights that although emotion verification enhances security by rejecting impostors, it also reduces usability by rejecting genuine users more frequently.

The score fusion-based approach effectively addresses the limitations of the two-step method by integrating emotion and speaker verification scores; however, achieving a significant reduction in EER compared to the conventional method, which relies solely on speaker verification. All three emotion verification models (ECAPA-TDNN, wav2vec 2.0, and HuBERT) demonstrate improved performance, with EERs of 1.34%, 1.30%, and 1.13%, respectively, compared to the conventional method’s 1.60% (Table 6). Among these, HuBERT yields the best performance at an optimal weight of α = 0.9 (Table 7), achieving an average EER of 1.13%. Although the EER increases with lower α values, the results show that emotion validation, when properly weighed, still contributes to accuracy.

Additionally, the superior performance of sadness and anger compared to neutral highlights the importance of leveraging their distinct acoustic features to enhance the robustness of emotion-dependent verification systems. The lower EERs observed with sadness (0.82%) and anger (0.88%), compared to neutral and joy (1.05 and 1.75%, respectively), further validate that these emotions enhance verification accuracy more effectively.

Extending this analysis, evaluations against spoofed speech with α = 0.8 reveal that the score fusion-based method maintains a lower average EER of 17.7% compared to the conventional method’s 25.1%, with sadness achieving the best performance at 14.2%. The EER for anger rises to 18.7%, however, suggesting that its acoustic features, while effective in non-spoofed contexts, may be more vulnerable to synthesis techniques. This reinforces the advantage of emotional diversity in strengthening security, particularly with sadness.

Considering the user experience, emotional speech requires more cognitive and physical effort than does neutral speech, particularly for anger and joy, which require higher vocal energy. In contrast, sadness, with tonal similarity to neutral, was the least effortful, aligning with its high verification performance. The increasing acceptance of the emotion-dependent system (Figure 9b) compared to the consistently high approval of the conventional system (Figure 9a) indicates, however, that while initial adaptation is needed, the proposed system is viable for long-term use with proper training. A limitation of the user evaluation experiment is the small and homogeneous sample of eight male students, however, which may reduce validity, as different results could potentially be obtained from female speakers or a more diverse population. Future experiments will address this by including a broader participant pool, including female speakers, to enhance the generalizability of researchers’ findings.

The experiment’s findings also underscore the robustness of the system, particularly its ability to effectively compare the acoustic characteristics of spontaneously expressed emotions rather than requiring trained or exaggerated acting. The average verification scores remaining above 0.5 for all emotions, even the more variable joy, demonstrate that the score fusion approach can leverage self-directed emotional expressions to enhance speaker verification accuracy. The high consistency of sadness (scores near 1.0) and its low perceived effort suggest that emotions with stable acoustic features can serve as reliable authentication keys, reducing the cognitive burden while maintaining security.

## 5. Conclusions

This study demonstrates that the emotion-dependent speaker verification system, enhanced by the score fusion-based approach, offers significant improvements in authentication robustness and usability compared to conventional methods. By leveraging the HuBERT model for emotion embeddings, the score fusion method achieved an average equal error rate (EER) of 1.13% for the speaker verification task, outperforming the conventional speaker verification system, which recorded an EER of 1.60%. This improvement stems from the effective integration of speaker and emotion information, highlighting the efficacy of the proposed framework.

In the anti-spoofing task, the emotion-dependent system further highlighted its robustness, reducing the FAR-FE to 1.5% against synthetic speech generated by the seed-VC model, compared to 17.6% for the baseline system relying solely on speaker embeddings. This enhanced resistance to spoofing attacks underscores the advantage of requiring both speaker identity and emotional consistency, making it significantly more challenging for impostors to succeed. Notably, sadness emerged as the optimal emotion for verification, offering low user effort, high emotion score stability (consistently near 1.0), and robust performance, challenging the conventional preference for neutral speech and supporting the system’s practical applicability.

Despite these advancements, the proposed method still exhibits limitations, as the EER for spoofed speech remains relatively high at 17.7%, and the user evaluation experiment is constrained by a small sample size of only eight male participants, exclusively focusing on Japanese speech. This may limit the model’s generalizability across different languages and diverse groups of speakers. In the future, more effective solutions are needed to better integrate speaker and emotion information, such as considering identification DNNs as an alternative to cosine similarity, as well as addressing speaker variation in emotional speech. Future work will also prioritize expanding the evaluation with a larger, more diverse participant pool and testing the model across multiple languages to improve its generalizability and practical applicability. These efforts will be crucial to further enhance the system’s security and adaptability for real-world deployment.

## Figures and Tables

**Table 1:** Sample of speech content of each emotion in JTES.

Emotion	Samples of Speech Content
Anger	今回は見逃すけど2度目はないからな (I will let it pass this time, but it will not happen again) 甘ったれるなよ (Do not be a spoiled brat) 何度思い出しても忌々しい (It is abhorrent no matter how many times I think about it)
Joy	今日は珍しく空いている (I am having a rare day off today) スペシャルフィギュア一発取りできた (I got a special figure in just one try) デート日和ですね (It is an enjoyable day for a date)
Sadness	今日は珍しく空いている (The pixel count is much lower than the latest models, so it is hard to get a good shot) 行きたいのは山々だけどちょっと財布と相談かな (As much as I would love to go, I am not sure I can afford it) 気温の変化が激しいから体調崩れっぱなしだ (With such rapid temperature changes, I am likely to get sick)
Neutral	黒髪サラサラロングの女の子です (She has long, silky black hair) 一番線に各駅停車代々木上原行きが来る (The train to Yoyogi Uehara arrives at Platform 1 soon) 環境構築は先輩に聞くのが手っ取り早い (The quickest way to build an environment is to ask a senior staff member)

**Table 2:** Sample of utterance content of each emotion in JTES.

Emotion	Utterance Content	Number of Speakers
Anger	あなたのせいで台無しだ (You have ruined it.)	5
	お前のせいで台無しだよ (You screwed this up.)	1
	お前のせいで台無しだ (You messed everything up.)	1
	なにやってんだよ (What the hell are you doing?)	1
Joy	チケット当選した (I won a ticket!)	5
	チケットに当選した (I have won a ticket!)	1
	一等当たった (I won the first prize!)	1
	よっしゃー、俺の勝ち〜 (I have got it! I have won!)	1
Sadness	抽選外れちゃった (I missed the lottery.)	6
	ガチャ大爆死した (I have missed all the gacha.)	1
	試験落ちたわ (I failed the exam.)	1
Neutral	昨日はバイトでした (I had a part-time job yesterday.)	6
	おはようございます (Good morning.)	1
	昨日は映画を見ました (I watched a movie yesterday.)	1

**Table 3:** Results of emotion verification by threshold comparison [%].

Target Speaker’s Utterances					
	Emotion				Average
	Anger	Joy	Sadness	Neutral	
FRR-TE	34.95	38.57	26.15	29.50	32.29
FAR-FE	34.99	38.57	26.17	29.54	32.33
Impostor’s Utterances					
	Emotion				Average
	Anger	Joy	Sadness	Neutral	
FRR-TE	100.00	100.00	100.00	99.99	99.99
FAR-FE	0.00	0.00	0.00	0.00	0.00

**Table 4:** Results of emotion verification by SVM [%].

Target Speaker’s Utterances					
	Emotion				Average
	Anger	Joy	Sadness	Neutral	
FRR-TE	31.85	47.97	28.07	45.83	38.43
FAR-FE	4.20	5.39	2.90	2.34	3.71
Impostor’s Utterances					
	Emotion				Average
	Anger	Joy	Sadness	Neutral	
FRR-TE	90.01	84.13	82.64	92.47	87.31
FAR-FE	5.44	11.14	9.01	4.81	7.60

**Table 5:** Results of speaker verification only [%].

	Emotion				Average
	Anger	Joy	Sadness	Neutral	
EER	1.28	2.16	1.19	1.76	1.60

**Table 6:** EER of conventional method and score fusion-based method using various emotion verification models [%].

	Emotion Verification Model	Emotions				Average
		Anger	Joy	Sadness	Neutral	
Conventional method	-	1.28	2.16	1.19	1.76	1.60
Score fusion-based method	ECAPA-TDNN	1.04	1.84	1.07	1.40	1.34
	wav2vec 2.0	0.87	2.00	0.86	1.45	1.30
	HuBERT	0.88	1.75	0.82	1.05	1.13

**Table 7:** EER of the proposed method using HuBERT for emotion verification at each value of the score-integration weight α [%].

Weight (α)	Emotion				Average
	Anger	Joy	Sadness	Neutral	
1.0	1.28	2.16	1.19	1.76	1.60
0.9	0.88	1.75	0.82	1.05	1.13
0.8	2.02	3.27	1.34	1.99	2.16
0.7	4.41	7.38	2.11	4.66	4.64
0.6	6.50	14.04	2.46	6.86	7.47
0.5	8.67	17.71	2.87	9.12	9.59

**Table 8:** EER of the score-fusion speaker verification method (α = 0.8) and conventional method against spoofed speech [%].

	Emotions				Average
	Anger	Joy	Sadness	Neutral	
Conventional method	27.9	28.5	19.1	25.1	25.1
Score fusion-based method	18.7	19.9	14.2	17.9	17.7

**Table 9:** FAR-FE of the score-fusion speaker verification method (α = 0.8) and conventional method against spoofed speech [%].

	Emotions				Average
	Anger	Joy	Sadness	Neutral	
Conventional method	22.5	22.7	10.0	15.3	17.6
Score fusion-based method	2.3	1.8	0.7	1.1	1.5

## Data Availability

The original contributions presented in this study are included in the article. Further inquiries can be directed to the corresponding author.

## Abbreviations

The following abbreviations are used in this manuscript:

EER	Equal Error Rate
FRR	False Rejection Rate
FAR	False Acceptance Rate
TE	True Emotion
FE	False Emotion
SVM	Support Vector Machine
